# Hypophosphatemia on ICU Admission Is Associated with an Increased Length of Stay in the ICU and Time under Mechanical Ventilation

**DOI:** 10.3390/jcm11030581

**Published:** 2022-01-24

**Authors:** Hannah Wozniak, André Dos Santos Rocha, Tal Sarah Beckmann, Christophe Larpin, Niccolò Buetti, Hervé Quintard, Jérôme Pugin, Claudia Paula Heidegger

**Affiliations:** 1 Division of Intensive Care, Department of Acute Medicine, Geneva University Hospitals, 1205 Geneva, Switzerland; christophe.larpin@hcuge.ch (C.L.); herve.quintard@hcuge.ch (H.Q.); jerome.pugin@hcuge.ch (J.P.); 2 Division of Anesthesiology, Department of Acute Medicine, Geneva University Hospitals, 1205 Geneva, Switzerland; andre.rocha@hcuge.ch (A.D.S.R.); tal.beckmann@hcuge.ch (T.S.B.); 3 Infection Control Program, World Health Organization Collaborating Centre on Patient Safety, Faculty of Medicine, 1205 Geneva, Switzerland; niccolo.buetti@hcuge.ch

**Keywords:** hypophosphatemia, metabolism, mechanical ventilation, ICU length of stay, COVID-19

## Abstract

Hypophosphatemia is frequently observed in the ICU and is associated with several impairments such as respiratory failure or infections. We hypothesized that hypophosphatemia on ICU admission is associated with a prolonged duration of mechanical ventilation and ICU length of stay (LOS), particularly in COVID-19 patients. This cross-sectional study analyzed data from 1226 patients hospitalized in the ICU of the Geneva University Hospitals from August 2020 to April 2021. Patients were categorized as having hypophosphatemia (phosphatemia ≤ 0.8 mmol/L) or non-hypophosphatemia (phosphatemia > 0.8 mmol/L) on ICU admission. Linear regressions were performed to investigate the association between hypophosphatemia on ICU admission and ICU LOS and duration of mechanical ventilation. Overall, 250 (20%) patients presented hypophosphatemia on ICU admission. In the univariable analysis, hypophosphatemic patients had longer ICU LOS than non-hypophosphatemic patients, 7.4 days (±10.4) versus 5.6 days (±8.3), (*p* < 0.01). Hypophosphatemia on ICU admission was associated with a prolonged duration of mechanical ventilation, 7.4 days (±11.2) versus 5.6 days (±8.9), (*p* < 0.01). These associations were confirmed in the multivariable analysis (*p* < 0.01). In the subgroup of COVID-19 patients, a significant association between hypophosphatemia and ICU LOS and duration of mechanical ventilation was also observed. In conclusion, hypophosphatemia on ICU admission is associated with a longer ICU LOS and time under mechanical ventilation, both in the general ICU population and in COVID-19 patients.

## 1. Introduction

Phosphate is an essential electrolyte involved in many physiological processes, including plasmatic acid–base buffering; preservation of cell membrane integrity, DNA, RNA, and protein metabolism; energy storage (high-energy bonds of adenosine triphosphate, ATP); and bone mineralization [1,2]. Hypophosphatemia (hypoP) can result from decreased intestinal absorption, increased renal excretion, lack of intake, or intra-cellular shifts [3], leading to systemic severity-related abnormalities, including neurological impairments (seizures, altered mental status), respiratory failure due to muscle weakness, decreased myocardial contractility, arrhythmias, hematological disorders, digestive dysfunction, and rhabdomyolysis [1,4]. Moreover, low blood phosphate levels may underestimate the extent of phosphate depletion, as only 1% of body phosphate is found in plasma [5]. Thus, reduced serum phosphate levels are usually the reflection of an important whole-body deficiency and/or an impairment of phosphate homeostasis [5]. Hypophosphatemia is found in up to 5% of hospitalized patients [2], with a prevalence of 20–64% in the intensive care unit (ICU) depending on the laboratory definition (range <0.32 to <0.8 mmol/L) [5].

An association between hypoP and length of mechanical ventilation, morbidity, and mortality has been suggested in the literature, with conflicting results and a lack of adjustment of confounding factors [4,6,7,8,9]. In addition, a link between low phosphate levels and the severity of COVID-19 has been suggested recently [10,11]. Since COVID-19 patients are currently an important part of ICU patients and are under mechanical ventilation for a long time, the prevalence and role of hypoP in this specific population should be investigated. Therefore, the objectives of the present study were to investigate the association between hypoP on ICU admission and ICU length of stay, duration of mechanical ventilation, and mortality in the general ICU population and in the COVID-19 sub-population.

## 2. Materials and Methods

This cross-sectional study was performed in the ICU of the Geneva University Hospitals. It is a mixed medical–surgical ICU admitting patients with a wide range of illnesses, including general, cardiovascular, and neurologic diseases and polytrauma patients, with nearly 2500 admissions per year. Over an 8-month study period, from the first of August 2020 to the first of April 2021, every patient admitted to the ICU was screened for participation in the study. Non-inclusion criteria were: age < 18 years old, chronic dialysis, and the lack of measurement of phosphatemia on ICU admission.

The following variables were collected on ICU admission from medical records in the hospital’s storage software: gender, age, BMI, APACHE II and SAPS II scores, phosphatemia, calcemia, eGFR (estimated by the Cockcroft–Gault equation), hospitalization for SARS-CoV2 pneumoniae, infection at admission or in the first 48 h following the admission, and reason for ICU admission (details of the reasons for admissions can be found in the Supplementary Materials). In addition, the following variables were recorded at the end of the ICU stay: intubation during ICU stay, new infection during ICU stay, presence of continuous veno-venous hemodiafiltration, ICU length of stay (LOS), and death. For mechanically ventilated patients, information about time under mechanical ventilation, weaning failure (defined as a need for reintubation in the first 48 h following extubation [12]), and new tracheotomy were also collected.

In case of several phosphatemia measurements in the first 24 h following ICU admission, the lowest value was collected. Patients were divided into two groups according to their phosphate levels on ICU admission: (1) hypophosphatemia (hypoP) was defined as phosphatemia ≤ 0.8 mmol/L [6,13,14] and (2) non-hypophosphatemia (non-hypoP) was defined as phosphatemia > 0.8 mmol/L. Our primary outcomes were ICU LOS and duration of mechanical ventilation. Our secondary outcome was mortality in the ICU.

The data collection was performed on the Redcap™ software. The study was approved by the authorities of the Geneva Ethics Committee (BASEC 2021-00594).

### Statistical Analysis

We performed descriptive analyses of patients’ characteristics on admission according to the phosphatemia status on ICU admission (hypoP or non-hypoP). Continuous variables were presented as mean ± 1 standard deviation (SD), and categorical variables were expressed as the number of patients (*n*) and percentage (%). Chi-square tests were used to detect differences in categorical variables and *t*-tests in continuous variables. All assumptions were met for normality. A univariable and multivariable linear regression was performed to characterize the association between hypoP at admission and the ICU length of stay (LOS) or duration of mechanical ventilation. As these last two variables were judged to have a log-normal distribution, log-transformed ICU LOS and days under mechanical ventilation were used for the statistical analysis. The following clinically relevant variables were determined a priori as potential confounding factors and were included in the multivariable analysis: gender, SAPS II score, reason for ICU admission, and the presence of new infection in the first 48 h following ICU admission [4,5,6,15]. To investigate the association between hypoP and mortality, we performed a multivariate logistic regression, adjusting the estimates for SAPS II, new infection in the first 48 h following ICU admission, gender, intubation, and reason for ICU admission. A subgroup analysis was performed for patients admitted to the ICU for COVID-19. Results of the linear regression are expressed as regression coefficient (coeff.) on a logarithmic scale with 95% confidence interval (CI 95%). Results of the logistic regression are expressed as odds ratios (OR) and 95% confidence interval (CI 95%). Two-tailed *p*-values ≤0.05 were considered statistically significant.

All statistical analyses were conducted using STATA version 16.1 (Stata Corp., College Station, TX, USA, 2007).

## 3. Results

### 3.1. Study Population Characteristics

**Figure 1:**
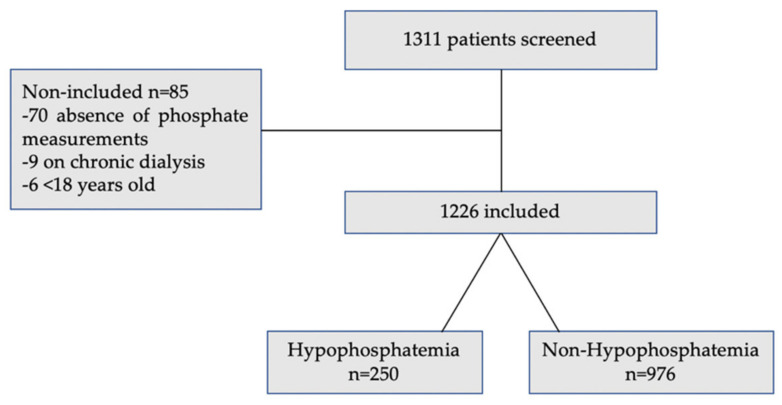
Study flowchart.

In total, 1311 patients were screened and 1226 were included in the analysis (Figure 1). The study population characteristics and the differences between hypophosphatemic and non-hypophosphatemic patients are presented in Table 1. Among them, 250 patients (20.3%) presented with hypoP (phosphatemia ≤ 0.8 mmol/L), including 67 COVID-19 patients (26.8%).

There were no differences in the mean APACHE II and mean SAPS II scores between hypoP and non-hypoP patients. Seventy percent of patients (856/1226) received invasive mechanical ventilation during ICU stay. In intubated patients, the duration of mechanical ventilation was significantly longer in hypoP patients than in non-hypoP patients, with 7.4 days (±11.2) and 5.6 days (±8.9) of mechanical ventilation, respectively (*p* = 0.02). There was no evidence of a statistical difference between patients who had hypophosphatemia and those who did not at the time of admission regarding weaning failure. Regarding ICU LOS, hypoP patients presented a longer LOS than non-hypoP patients, with 7.4 (±10.4) and 5.6 (±8.3) days in the ICU, respectively (*p* < 0.01). No difference in mortality was observed between the two groups.

Characteristics and differences between COVID-19 and non-COVID-19 patients are presented in Appendix A, Table A1. The prevalence of hypophosphatemia on ICU admission was 26.3% and 18.9% in COVID-19 and non-COVID-19 patients, respectively (*p* < 0.01).

### 3.2. Hypophosphatemia and Duration of Mechanical Ventilation

**Figure 2:**
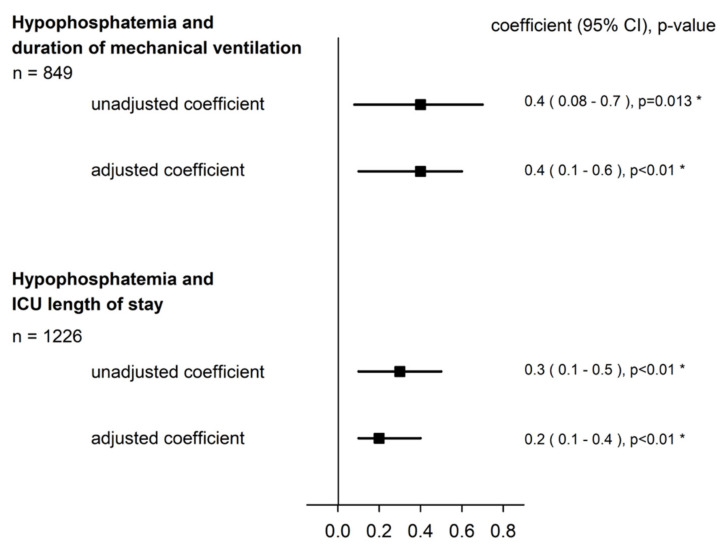
Hypophosphatemia, duration of mechanical ventilation, and ICU LOS in all ICU patients. Regression coefficient (95% confidence interval) on a logarithmic scale. When the log-transformed coefficient was back-transformed (i.e., exponentiated), hypoP at ICU admission multiplied the duration of mechanical ventilation by 1.5 days (CI 95%: 1.1–1.8) and the ICU LOS by 1.2 days (CI 95%: 1.1–1.5). Adjusted coefficient was adjusted to: gender, SAPSII on ICU admission, infection within the first 48 h, and reason for ICU admission.*: *p* < 0.05.

**Figure 3:**
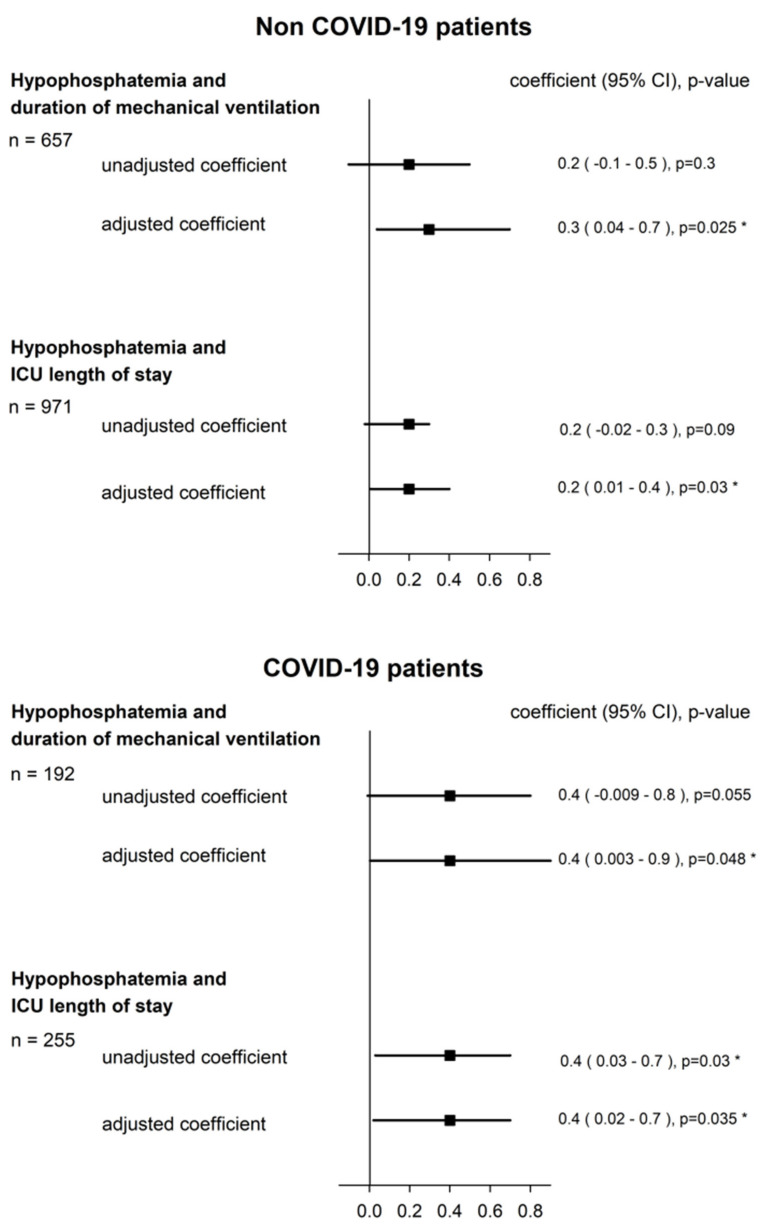
Hypophosphatemia, duration of mechanical ventilation, and ICU LOS in non-COVID-19 patients and COVID-19 patients. Regression coefficient (95% confidence interval) on a logarithmic scale. Adjusted coefficient was adjusted to: gender, SAPSII on ICU admission, infection within the first 48 h, and reason for ICU admission (COVID-19 patients were not further adjusted for reason for ICU admission.). *: *p* < 0.05.

The univariable and multivariable linear regression showed a significant association between hypoP and duration of mechanical ventilation (Figure 2; Appendix A, Table A2). When the log-transformed coefficient was back-translated (i.e., exponentiated) for ease of interpretation, hypoP at ICU admission multiplied the duration of mechanical ventilation by 1.5 days (CI 95%: 1.1–1.8). In addition, a significant association was also found in the subgroup analysis of COVID-19 patients and non-COVID-19 patients (Figure 3; Appendix A, Table A3).

### 3.3. Hypophosphatemia and ICU Length of Stay

A significant association between hypoP at admission and ICU LOS was also observed in the univariable and multivariable model (Figure 2; Appendix A, Table A4). When the log-transformed coefficient was back-translated (i.e., exponentiated) for ease of interpretation, hypoP at admission multiplied the ICU LOS by 1.2 days (CI 95%: 1.1–1.5). In the subgroup analysis of COVID-19 patients and non-COVID-19 patients, a significant association with a longer ICU LOS was also observed (Figure 3; Appendix A, Table A5).

### 3.4. Hypophosphatemia and Mortality in the ICU

There was no evidence for a statistical association between hypoP on ICU admission and mortality in the ICU in the multivariate logistic regression (Table 2).

## 4. Discussion

We report data from 1226 ICU patients, including 255 patients with SARS-CoV2 pneumonia, demonstrating an association between hypophosphatemia (≤0.8 mmol/L) on ICU admission and time under mechanical ventilation and length of stay in the ICU. No association was found between hypoP at ICU admission and all-cause mortality.

A possible explanation for the longer duration of mechanical ventilation observed in patients with hypoP could be that phosphate is a key element for the biosynthesis of ATP, which is produced in large quantities by respiratory muscles, including the diaphragm. Therefore, it is likely that a deficiency in phosphate is associated with diaphragmatic dysfunction, leading to an increased mechanical ventilation time. A previous study on patients with chronic obstructive pulmonary disease (COPD) supports this hypothesis, showing that in the case of hypoP, COPD patients presented signs of weakness of the respiratory muscles, resulting in reduced tidal volume [16]. Previous studies of the association between hypoP on ICU admission and the length of mechanical ventilation generated conflicting results [5,7,13,15,17]. This may be due to small size, underpowered studies. We believe that the large sample size of the present study yielded the necessary statistical power to demonstrate a significant association and indicate that hypoP on ICU admission is a good indicator for the duration of mechanical ventilation.

In line with previous studies [6,7,15], our study demonstrated that hypoP on ICU admission was associated with a prolonged ICU LOS. The relationship between hypoP, ICU LOS, and time on the ventilator is particularly relevant since hypoP occurs in 20–64% of patients admitted to the ICU [5,14]. In addition to the effect of hypoP on respiratory muscles’ strength, phosphate plays a role in many physiological processes, and its deficiency can lead to various impairments such as decreased cardiac contractility, arrhythmias, and neurologic disorders [3,18]. The longer ICU LOS observed in patients with hypoP could, therefore, be related to prolonged mechanical ventilation but also to an increased number of these complications.

Recent studies have reported that hypoP might be associated with all-cause mortality in the ICU [6,7]. A recent meta-analysis totaling more than 7000 ICU patients did not find such an association [4]. Similarly, such an association between hypoP and mortality was not found in the present study. This could be explained in part by the low ICU mortality rate of 12.7% found in our study, which could have resulted in a lack of power to demonstrate such an association. Herein, only ICU mortality was studied; perhaps hypoP may be associated with 90-day mortality or hospital mortality. Interestingly the studies in which such an association was found studied 28-day and 180-day mortality [6,7]. Finally, one cannot exclude that hypoP may only be a marker of disease severity and, therefore, not directly associated with mortality [4,15].

With the ongoing COVID-19 pandemic, SARS-COV2 patients have been continuously occupying ICU beds since February 2020. Phosphatemia in this particular population has been scarcely studied [10,11,19]. Phosphatemia levels may be lower in critical COVID-19 patients, as these patients tend to be older and more likely to be obese and malnourished, known risk factors for hypoP [10]. Indeed, our study found that COVID-19 patients had a higher prevalence of hypophosphatemia compared with non-COVID-19 patients. In the COVID-19 population, hypoP was also associated with a longer ICU stay and mechanical ventilation duration. A previous study conducted in a small group of patients (*n* = 32) found that patients with severe COVID-19 pneumonia had lower phosphate levels than less severe patients [19]. Another study conducted on few non-ICU patients (*n* = 36) showed that patients with hypoP had more severe lung damage than those with normal or high phosphate levels [11]. It has been hypothesized that hypoP and ATP depletion could overactivate the immune system, thereby promoting a cytokine storm and acute respiratory distress syndrome (ARDS) [10]. Therefore, hypoP might be a good severity marker in COVID-19 patients upon admission to the ICU.

Some limitations in our study need to be acknowledged. The cross-sectional design does not allow us to infer causality between hypoP and ICU LOS and mechanical ventilation days but solely to describe an association. Although the hypoP measured in the blood only reflects low extracellular phosphate levels, which represent 1% of the total body phosphate level, plasma hypoP seems to reveal a regulatory disorder and/or a body phosphate depletion [5]. We did not perform a predictive model between phosphate level on ICU admission as a continuous variable and the study outcomes since we did not expect a linear association; therefore, we categorized the phosphate levels in hypoP and non-hypoP. Hyperphosphatemia was not assessed in our study. In addition, the cause and the treatment of hypoP could not be assessed in our study. Our study focused on phosphatemia on ICU admission, and we did not investigate whether phosphate substitution was carried out or whether this affected the outcome of ICU LOS and mechanical ventilation. Since phosphate substitution can be carried out easily, quickly, and without risk to the patient, further studies are warranted to investigate whether phosphate substitution can influence the patient outcome, as only few low-quality studies have so far focused on this point without any conclusive result [20,21].

## 5. Conclusions

This study highlights the value of plasma phosphate levels measured on ICU admission as an early indicator of ICU length of stay and duration of mechanical ventilation in both the general ICU population and in the subgroup of COVID-19 patients. Further studies should focus on phosphate substitution and clinical outcomes of ICU patients.

## Figures and Tables

**Table 1:** Characteristics and differences between hypophosphatemic and non-hypophosphatemic critically ill patients.

	Total *n* = 1226	Non-HypoP (>0.8 mmol/L) *n*= 976	HypoP (≤0.8 mmol/L) *n* = 250	*p* Value
**Patient characteristics**				
Gender male, *n* (%)	807 (65.8%)	640 (65.6%)	167 (66.8%)	0.7
Age, mean (SD)	61.2 (15.1)	61.6 (15.2)	59.8 (14.7)	0.09
BMI, mean (SD)	26.8 (7)	26.8 (6.5)	27.1 (9)	0.6
APACHE II, mean (SD)	24.2 (8.6)	24.4 (8.7)	23.4 (8.2)	0.1
SAPS II, mean (SD)	50.6 (20)	50.9 (20.3)	49.4 (18.4)	0.3
Reason for ICU admission, *n* (%)				<0.01 *
- Septic shock	83 (6.8%)	67 (6.9%)	16 (6.4%)	
- Cardiovascular disease	301 (24.6%)	253 (26%)	48 (19.2%)	
- Respiratory failure	300 (24.4%)	224 (23%)	76 (30.3%)	
- Neurological disease	335 (27.3%)	258 (26.4%)	77 (30.8%)	
- Abdominal disease	148 (12.1%)	129 (13.2%)	19 (7.6%)	
- Others (hematologic, ENT, or metabolic disease)	59 (4.8%)	45 (4.6%)	14 (5.6%)	
eGFR <60 mL/min at ICU admission, *n* (%)	354 (28.9%)	319 (32.8%)	35 (14.1%)	<0.01 *
Phosphatemia on ICU admission, mmol/L mean (SD)	1.12 (0.46)	1.3 (0.4)	0.6 (0.1)	<0.01 *
Ionized calcemia on ICU admission, mmol/L mean (SD)	1.14 (0.1)	1.15 (0.1)	1.13 (0.09)	<0.01 *
Intubated patients during ICU stay, *n* (%)	856 (69.8%)	670 (68.7%)	186 (74.4%)	0.08
Weaning ventilator failure, *n* (%)	91 (10.6%)	75 (11.2%)	16 (8.6%)	0.5
New tracheotomy during ICU stay, *n* (%)	49 (4%)	35 (3.6%)	14 (5.6%)	0.1
Infections during the first 48 h of ICU stay, *n* (%)	526 (42.9%)	414 (42.4%)	112 (44.8%)	0.5
New infections during ICU stay, *n* (%)	48 (3.9%)	33 (3.4%)	15 (6%)	0.06
Hemodiafiltration during ICU stay, *n* (%)	50 (4.1%)	44 (4.5%)	6 (2.4%)	0.1
COVID-19 patients, *n* (%)	255 (20.8%)	188 (19.3%)	67 (26.8%)	<0.01 *
**Study outcomes**				
Ventilation time in days, mean (SD)	6 (9.5)	5.6 (8.9)	7.4 (11.2)	0.02 *
ICU length of stay, mean (SD)	6 (8.8)	5.6 (8.3)	7.4 (10.4)	<0.01 *
Death in the ICU, *n* (%)	156 (12.7%)	126 (12.9%)	30 (12%)	0.7

Two-sided Pearson’s chi-squared test for categorical variables, *t*-test for continuous variable. * *p* < 0.05. HypoP, hypophosphatemia (defined as phosphatemia ≤ 0.8 mmol/L); APACHE II, Acute Physiology and Chronic Health Evaluation 2; SAPS II, Simplified Acute Physiology Score II; ENT, ear, nose, and throat diseases.

**Table 2:** Hypophosphatemia and mortality in the ICU.

*n* = 1226	Mortality in the ICU, Odds Ratio (95% CI)	*p* Value
Hypophosphatemia	1.09 (0.69–1.72)	0.7
Gender male	1.55 (1.02–2.35)	0.04 *
SAPS II	1.05 (1.04–1.07)	<0.01 *
Infections within the first 48 h	2.14 (1.46–3.13)	<0.01 *
Intubation	1.68 (0.89–3.15)	0.11
Reason for ICU admission		<0.01 *
- Abdominal disease	1.0 (Reference)	NA
- Septic shock	1.32 (0.5–3.5)	0.57
- Cardiovascular disease	2.85 (1.31–6.21)	<0.01 *
- Respiratory failure	3.56 (1.68–7.54)	<0.01 *
- Neurological disease	2.83 (1.30–6.17)	<0.01 *
- Others (hematologic, ENT, or metabolic disease)	0.63 (0.13–3.07)	0.56

SAPS II, Simplified Acute Physiology Score II; ENT, ear, nose, and throat diseases. * *p* < 0.05.

## Data Availability

The datasets used and analyzed during the current study are available from the corresponding author on reasonable request.

## Supplementary Materials

The following are available online at www.mdpi.com/article/10.3390/jcm11030581/s1, File S1: Reason for ICU admission based on the diagnosis on ICU admission.

## Appendix A

**Table A1:** Characteristics and differences of critically ill patients hospitalized in the ICU for non-COVID-19 and COVID-19.

	Total *n* = 1226	Non-COVID-19 *n* = 971	COVID-19 *n* = 255	*p* Value
**Patient characteristics**				
Gender male, *n* (%)	807 (65.8%)	620 (63.9%)	187 (73.3%)	<0.01 *
Age, mean (SD)	61.2 (15.1)	60.5 (0.5)	64 (0.7)	<0.01 *
BMI, mean (SD)	26.8 (7)	26.1 (0.2)	29.7 (0.6)	<0.01 *
APACHE II, mean (SD)	24.2 (8.6)	24.3 (8.5)	23.7 (9.1)	0.3
SAPSII, mean (SD)	50.6 (20)	49.7 (0.6)	53.8 (0.6)	<0.01 *
eGFR <60 mL/min at ICU admission, *n* (%)	354 (29%)	305 (31.4%)	49 (19.2%)	<0.01 *
Phosphatemia on ICU admission, mmol/L mean (SD)	1.12 (0.46)	1.2 (0.02)	1.02 (0.1)	<0.01 *
Prevalence of hypophosphatemia on ICU admission, mean (SD)	250 (20.4%)	183 (18.9%)	67 (26.3%)	<0.01 *
Ionized calcemia on ICU admission, mmol/L mean (SD)	1.14 (0.1)	1.15 (0.03)	1.11 (0.005)	<0.01 *
Intubated patients during ICU stay, *n* (%)	856 (69.8%)	664 (68.4%)	192 (75.3%)	<0.01 *
Weaning ventilator failure, *n* (%)	91 (10.6%)	61 (6.3%)	30 (11.8%)	<0.01 *
New tracheotomy during ICU stay, *n* (%)	49 (4%)	24 (2.5%)	25 (9.8%)	<0.01 *
Infections during the first 48 h of ICU stay, *n* (%)	526 (42.9%)	293(30.1%)	233 (91.4%)	<0.01 *
New infections during ICU stay, *n* (%)	48 (3.9%)	42 (4.3%)	6 (2.4%)	0.14
Hemodiafiltration during ICU stay, *n* (%)	50 (4.1%)	43 (4.4%)	7 (2.8%)	0.2
**Study outcomes**				
Ventilation time in days, mean (SD)	6 (9.5)	3.8 (6.4)	13.2 (13.7)	<0.01 *
ICU length of stay, mean (SD)	6 (8.8)	4.4 (6.4)	12 (13)	<0.01 *
Death in the ICU, *n* (%)	156 (12.7%)	96 (9.9%)	60 (23.5%)	<0.01 *

Two-sided Pearson’s chi-squared test for categorical variables, *t*-test for continuous variable. * *p* < 0.05. APACHE II, Acute Physiology and Chronic Health Evaluation 2; SAPS II, Simplified Acute Physiology Score II; ENT, ear, nose, and throat diseases.

**Table A2:** Hypophosphatemia and time under mechanical ventilation.

**Univariable Analysis, *n* = 849**	**Days of Mechanical Ventilation Regression Coefficient, (95% CI)**	***p* Value**
Non-hypophosphatemia	Ref.	
Hypophosphatemia	0.4 (0.08–0.7)	0.013 *
**Multivariable Analysis, *n* = 849**	**Days of Mechanical Ventilation Regression Coefficient, (95% CI)**	***p* Value**
Non-hypophosphatemia	Ref.	
Hypophosphatemia	0.4 (0.1–0.6)	<0.01 *
Gender male	0.1 (−0.1–0.3)	0.4
SAPS II	0.03 (0.02–0.03)	<0.01 *
Infection within the first 48 h	0.8 (0.6–1.1)	<0.01 *
Reason for ICU admission		
- Abdominal disease	Ref.	NA
- Septic shock	0.2 (−0.4–0.7)	0.5
- Cardiovascular disease	−0.3 (−0.7–0.03)	0.07
- Respiratory failure	1.2 (0.8–1.6)	<0.01 *
- Neurological disease	0.2 (−0.2–0.5)	0.3
- Others (hematologic, ENT, or metabolic disease)	−0.2 (−0.8–0.3)	0.4

Regression coefficient (95% confidence interval) on a logarithmic scale. * *p* < 0.05. SAPSII, Simplified Acute Physiology Score II; ENT, ear, nose, and throat diseases.

**Table A3:** Duration of mechanical ventilation in non-COVID-19 and COVID-19 patients.

	Non-COVID-19 Patients (*n* = 657)		COVID-19 Patients (*n* = 192)	
**Univariable Analysis**	**Days of Mechanical Ventilation Regression Coefficient, (95% CI)**	***p***	**Days of Mechanical Ventilation Regression Coefficient, (95% CI)**	***p***
Non-hypoP (≥0.8 mmol/L)	Ref.		Ref.	
HypoP (<0.8 mmol/L)	0.2 (−0.1–0.5)	0.3	0.4 (−0.009–0.8)	0.055
**Multivariable Analysis**	**Days of Mechanical Ventilation, Regression Coefficient, (95% CI)**	***p***	**Days of Mechanical Ventilation, Regression Coefficient, (95% CI)**	***p***
Non-hypoP (≥0.8 mmol/L)	Ref.		Ref.	
HypoP (<0.8 mmol/L)	0.3 (0.04–0.7)	0.025 *	0.4 (0.003–0.9)	0.048 *
Gender male	0.09 (−0.2–0.4)	0.5	0.07 (−0.4–0.5)	0.7
SAPS II	0.04 (0.03–0.04)	<0.01 *	0.003 (−0.007–0.1)	0.6
Infections within the first 48 h	0.8 (0.5–1.1)	<0.01 *	0.9 (0.2–1.5)	<0.01 *
Reason for ICU admission				
- Abdominal disease	Ref.	NA		
- Septic shock	0.1 (−0.5–0.7)	0.7		
- Cardiovascular disease	−0.3 (−0.7–0.05)	0.09		
- Respiratory failure	0.4 (−0.2–0.9)	0.2		
- Neurological disease	0.2 (−0.1–0.6)	0.2		
- Others (hematologic, ENT or metabolic disease)	−0.2 (−0.8–0.3)	0.4		

Regression coefficient (95% confidence interval) on a logarithmic scale. * *p* < 0.05. SAPSII, Simplified Acute Physiology Score II; ENT, ear, nose, and throat diseases.

**Table A4:** Hypophosphatemia and ICU length of stay.

Univariable Analysis, *n* = 1226	ICU Length of Stay in Days Regression Coefficient, (95% CI)	*p* Value
Non-hypophosphatemia	Ref.	
Hypophosphatemia	0.3 (0.1–0.5)	<0.01 *
**Multivariable Analysis, *n* = 1226**	**ICU Length of Stay in Days Regression Coefficient, (95% CI)**	***p* Value**
Non-hypophosphatemia	Ref.	
Hypophosphatemia	0.2 (0.1–0.4)	<0.01 *
Gender male	0.03 (−0.1–0.2)	0.7
SAPS II	0.02 (0.01–0.02)	<0.01 *
Infections within the first 48 h	0.5 (0.3–0.6)	<0.01 *
Reason for ICU admission		
- Abdominal disease	Ref.	NA
- Septic shock	−0.09 (−0.4–0.2)	0.6
- Cardiovascular disease	−0.007 (−0.2–0.2)	0.9
- Respiratory failure	0.6 (0.4–0.8)	<0.01 *
- Neurological disease	0.1 (−0.8–0.3)	0.2
- Others (hematologic, ENT, or metabolic disease)	−0.05 (−0.3–0.3)	0.8

Regression coefficient (95% confidence interval) on a logarithmic scale. * *p* < 0.05. SAPSII, Simplified Acute Physiology Score II; ENT, ear, nose, and throat diseases.

**Table A5:** Length of stay in the ICU in non-COVID-19 and COVID-19 patients in days.

	Non-COVID-19 Patients (*n* = 971)		COVID-19 (*n* = 255)	
**Univariable Analysis**	**ICU Length of Stay in Days Regression Coefficient, (95% CI)**	***p***	**ICU Length of Stay in Days Regression Coefficient, (95% CI)**	***p***
Non-hypoP (≥0.8 mmol/L)	Ref.		Ref.	
HypoP (<0.8 mmol/L)	0.2 (−0.02–0.3)	0.09	0.4 (0.03–0.7)	0.03 *
**Multivariable Analysis**	**ICU Length of Stay in Days Regression Coefficient, (95% CI)**	***p***	**ICU Length of Stay in Days Regression Coefficient, (95% CI)**	***p***
Non-hypoP (≥0.8 mmol/L)	Ref.		Ref.	
HypoP (<0.8 mmol/L)	0.2 (0.01–0.4)	0.03 *	0.4 (0.02–0.7)	0.035 *
Gender male	−0.02 (−0.2–0.1)	0.8	0.07 (−0.2–0.4)	0.6
SAPS II	0.02 (0.01–0.02)	<0.01 *	0.01 (0.004–0.02)	<0.01 *
Infections within the first 48 h	0.4 (0.2–0.6)	<0.01 *	0.5 (0.02–1)	0.04 *
Reason for ICU admission				
- Abdominal disease	Ref.			
- Septic shock	−0.07 (−0.4–0.2)	0.7		
- Cardiovascular disease	−0.02 (−0.2–0.2)	0.8		
- Respiratory failure	−0.07 (−0.4–0.2)	0.6		
- Neurological disease	0.1 (−0.09–0.3)	0.2		
- Others (hematologic, ENT, or metabolic disease)	−0.07 (−0.4–0.2)	0.6		

Regression coefficient (95% confidence interval) on a logarithmic scale.* *p* < 0.05. SAPSII, Simplified Acute Physiology Score; ENT, ear, nose, and throat diseases. Regression coefficient (95% confidence interval) on a logarithmic scale.
