# Supplementary material for: Hypophosphatemia on ICU Admission Is Associated with an Increased Length of Stay in the ICU and Time under Mechanical Ventilation
Source: J Clin Med. 2022 Jan 24;11(3):581. doi: 10.3390/jcm11030581 (PMC8836766; doi:10.3390/jcm11030581)
Supplement: Supplementary file 1 [file jcm-11-00581-s001.zip › jcm-1555608-supplementary.pdf]

Supplementary Materials

Reason for ICU admission based on the diagnosis on ICU admission

1) Septic shock, including: admission for septic shock
2) Cardiovascular diseases, including: acute coronary syndrome, cardiac arrest, pulmonary embolism, heart failure, cardiogenic shock, abdominal aortic aneurysm, aortic dissection, rhythm disorder, post vascular or heart surgery, other cardiovascular disease
3) Respiratory failure, including: acute respiratory distress syndrome, exacerbation of chronic obstructive pulmonary disease, exacerbation of asthma, pneumonia without criteria of septic shock
4) Neurological disease, including: subarachnoid hemorrhage, traumatic craniocerebral injury, intracranial hemorrhage, stroke, epilepsy, cerebral neoplasia, post neurosurgery, other neurological disease
5) Abdominal disease, including: liver or biliary tract disease, digestive hemorrhage, intestinal obstruction, digestive perforation, renal or liver transplant, urogenital or digestive tract neoplasia, urogenital tract disease, other abdominal disease
6) Others, including: diabetes, drug intoxication, neoplasia of the ear, nose, throat (ENT) sphere, ENT disease, orthopedic surgery
